# Conditions Associated With the Onset of Cancer After Heart Transplant: Longitudinal Study in 335 Recipients

**DOI:** 10.1111/ctr.70243

**Published:** 2025-07-25

**Authors:** Fabian Patauner, Filomena Boccia, Silvia Masini, Giuliana Autiero, Raffaella Gallo, Lorenzo Bertolino, Irene Mattucci, Daniela Pinto, Roberto Andini, Cristiano Amarelli, Rosa Zampino, Emanuele Durante‐Mangoni

**Affiliations:** ^1^ Department of Precision Medicine University of Campania “Luigi Vanvitelli” Napoli Italy; ^2^ Department of Biomedical Sciences Humanitas University Milan Italy; ^3^ Department of Internal Medicine Azienda Ospedale Università Padova Padova Italy; ^4^ Department of Cardiac Surgery and Transplants A.O.R.N. Ospedali dei Colli ‐ Ospedale Monaldi Napoli Italy; ^5^ Unit of Internal Medicine & Transplants A.O.R.N. Ospedali dei Colli ‐ Ospedale Monaldi Napoli Italy; ^6^ Department of Advanced Medical and Surgical Sciences University of Campania “Luigi Vanvitelli Napoli Italy

**Keywords:** cancer, heart transplant, ischemic cardiomyopathy, neoplasia, risk factors

## Abstract

Cancer is among the major causes of death after heart transplant (HTx). Risk factors for cancer occurrence in this setting are not well established. This was a retrospective observational study of patients who underwent HTx between 2006 and 2019 and were followed up until May 2024. Clinical variables possibly associated with cancer were assessed with univariable and multivariable analyses. Survival analysis was carried out drawing Kaplan Meier curves and a Cox regression with time‐varying covariates were performed to overcome the immortal‐time‐bias. Three‐hundred‐thirty‐five HTx recipients were included, of whom 42 (12.5%) developed cancer after a median of 6.3 years. In univariable analysis, older age at HTx, smoking history, alcohol use, male sex, ischemic heart disease before HTx, use of cyclosporine rather than tacrolimus, and increased length of follow‐up were associated with cancer. Upon multivariable analysis, ischemic heart disease (OR 2.70 [1.19–6.11], *p* = 0.017) and length of follow‐up (OR 1.02 [1.00–1.04], *p* = 0.007) were independently associated with cancer occurrence. Cox regression revealed a higher risk of mortality among patients with cancer (HR 3.400, [2.026–5.709], *p* < 0.001). HTx recipients with prior ischemic cardiomyopathy and a longer survival time after transplant show a higher risk of developing cancer. Cancer significantly impairs post‐transplant survival.

**Abbreviations**
HTx: heart transplant
NMSC: non‐melanoma skin cancer
PRA: Panel Reactive Antibody
PTLD: post‐transplant lymphoproliferative disorders

## 1 Introduction

Cardiac transplantation is the treatment of choice for advanced heart failure refractory to medical and device therapy, when no absolute contraindications exist [1]. Heart transplant (HTx) recipients are immediately placed on immunosuppression to prevent acute rejection [2]. After an initial high‐intensity phase, the immune suppressive regimen is progressively attenuated to balance lower risks of rejection with increasing hazards of complications due to life‐long, cumulative exposure to chronic immunosuppression [3, 4]. Causes of death in HTx patients may vary over time, with graft failure, infections ad multiorgan failure more frequently observed during the first year and cancer, cardiac allograft vasculopathy (CAV), and renal failure becoming more incident with increasing post‐transplant years [2, 5, 6]. The overall rate of cancer among HTx recipients is estimated to be 15.3% [7]. Non‐melanoma skin cancer is the most common cancer observed after HTx, followed by post‐transplant lymphoproliferative disorders (PTLD); among solid tumors, lung cancer features prominently [8]. Reduction of immunosurveillance, reactivation of oncogenic viruses and carcinogenic effects of immunosuppressants are major risk factors [9]. However, many HTx candidates already present various comorbidities that share with cancer the same drivers, such as a history of cigarette smoking [9, 10].

Cancer occurrence in transplant recipients may be a serious challenge. Indeed, aggressive features, progression time and cancer mortality are all greater in this subgroup of patients [11, 12]. In addition, feasibility of chemotherapy remains low in these frail patients due to increased risks of serious adverse events, graft rejection, or worsened immune compromise [13]. Furthermore, specific screening programs for solid cancers in HTx recipients are lacking and recommendations mostly follow guidelines available for the general population [14]. Consequently, delays in cancer diagnosis may occur in transplant recipients [15].

Evaluation of correlations between clinical variables at the time of HTx and cancer occurrence may help identify specific potential cancer risk factors in these patients. Furthermore, whether cancer occurrence significantly influences recipient survival remains to be investigated. Accordingly, this study assessed several clinical features for their possible association with cancer occurrence in HTx recipients. Specifically, we studied the occurrence of cancer and the clinical variables associated with this complication in a large, single center cohort of HTx recipients, with the aim of identifying possible risk factors, allowing to tailor screening programs.

## 2 Material and Methods

### 2.1 Study Design

This is a retrospective observational analysis of clinical and biochemical data obtained from 335 HTx recipients. Included in the study were all consecutive patients who received the graft between January 2005 and December 2019 and were followed up until May 2024 in the AORN Ospedali dei Colli‐Ospedale Monaldi, Naples, Italy. Data were collected from electronic charts, available for each patient hospitalization or outpatient visit, filling in a dedicated case report form. Data analyzed included past medical history, exposure to known cancer risk factors, immunosuppressive therapy, clinical course after transplantation, and viral reactivations. According to the diagnosis of cancer after HTx, patients were divided in two groups and studied for the collected variables by univariable and multivariable models (see below).

This study was approved by our University Ethics Committee (protocol n. 28/2019) and was compliant with the 1975 Declaration of Helsinki and its later amendments. Due to the retrospective analysis of data, a specific patient informed consent was not required.

### 2.2 Analyzed Variables

Data collected included: sex, age at heart transplant, date of last established follow‐up, family history of cancer, alcohol consumption (defined as any amount of alcohol intake), cigarette smoking exposure (both prior and active at the time of data collection), occupational exposure to any known cancerogenic risk factor, Cytomegalovirus (CMV) mismatch (donor and receiver serostatus), Panel Reactive Antibody (PRA) status; pre‐transplant comorbidities such as diabetes, established metabolic syndrome, and systemic inflammatory diseases including both rheumatologic and non‐rheumatologic conditions were also included.

Post‐transplant complications, including acute cellular rejection, antibody‐mediated rejection, cardiac allograft vasculopathy, presence of donor‐specific antibodies (DSA) and CMV and EBV reactivations were also analyzed. In addition, data regarding immunosuppressive regimens were collected, although in patients with cancer occurrence, these refer only to the period between HTx and cancer diagnosis. CMV and EBV reactivations after HTx were defined as any positive result for viral nucleic acids in whole blood samples with real time polymerase chain reaction (CMV ELITe MGB kit, ELITech Group), regardless of viral load and clinical signs or symptoms of disease.

At the time of cancer diagnosis, the following data were analyzed: date of diagnosis; previous history of cancer; the time interval between HTx and cancer diagnosis; organ/site of onset and histology of cancer. Blood samples were collected according to the routine clinical follow‐up protocol for HTx patients. The follow‐up period was determined as days from the date of transplantation to the end of follow‐up with data on clinical status (alive or deceased), considering those who died and those who were lost to follow‐up over time.

### 2.3 Statistical Analysis

Categorical and nominal data were expressed as numbers and percentages, while numerical data were expressed as mean and standard deviation (SD) or as median with interquartile range (IQR). Fisher's exact test or chi‐square test were used to compare nominal variables. In contrast, numerical variables were analyzed with nonparametric Mann‐Whitney's U‐test or Kruskal‐Wallis test.

A *p* value <0.05 identified variables considered significantly associated with each outcome. All variables significantly associated to the outcome were included in a multivariable analysis to identify independent predictors of outcome using a stepwise backward conditional analysis. The binary logistic regression was adjusted for age at HTx, sex, and length of follow‐up with Inverse Probability Weighting (IPW). Missing data were excluded from analysis. Kaplan Meier curves for the overall cohort, and after excluding recipients with a follow up shorter than 1 year, were drawn for survival analysis. Cox regression with time varying covariates was performed to analyze cancer occurrence and mortality. In addition, considering the long time (2005–2019) in which patients received the graft, we stratified the cohort in five groups according to the year of HTx and then Kaplan Meier curve for cancer occurrence were drawn and time‐to‐event analysis was performed using log rank test. All statistical tests were computed using SPSS v. 29 (Armonk, NY: IBM Corp) and R v. 4.4.1 (R Development Core Team).

## 3 Results

Enrolled in this study were 335 HTx recipients. The median age at transplantation was 53 years and the majority were males (77.6%). Of the 335 patients included, 42 (12.5%) developed a cancer during the post‐transplant follow‐up, equal to an incidence of 16.1 cases per 1000 patient‐years. Death due to any cause occurred in 185 (55.2%) patients during follow‐up, of whom 83 died within 1 year of transplant. Clinical features of the study cohort are detailed in Table 1.

**TABLE 1:** Clinical features of the overall 335 heart transplant recipients studied and univariable and multivariable analyses.

Parameter	Overall cohort (*N* = 335)	Cancer (*N* = 42)	No cancer (*N* = 293)	*p* value	Multivariable analysis	
					OR [95% CI]	*p* value
Age at HTx, *years*	53 [42–59]	57 [51–62]	52 [40–59]	**0.006**	1.02 [0.98–1.06]	0.206
Sex, male	260 (77.6)	38 (90.5)	222 (75.8)	**0.031**	4.45 [0.97–20.38]	0.054
Family history of cancer	56 (16.7)	12 (28.6)	44 (15.0)	0.142		
Reason for HTx						
Ischemic heart disease	123 (36.7)	26 (61.9)	97 (33.1)			
Dilative cardiomyopathy	139 (41.5)	12 (28.6)	127 (43.3)			
Hypertrophic cardiomyopathy	25 (7.5)	1 (2.4)	24 (8.2)			
Valvular heart disease	25 (7.5)	2 (4.8)	23 (7.8)			
Others	23 (6.8)	1 (2.4)	22 (7.5)			
Prior ischemic heart disease	123 (36.7)	26 (61.9)	97 (33.1)	**<0.001**	2.70 [1.19–6.11]	**0.017**
Smoking history	172 (51.3)	33 (78.6)	139 (47.4)	**0.011**	1.23 [0.40–3.76]	0.715
Alcohol consumption	84 (25.1)	18 (42.9)	66 (22.5)	**0.043**		
Occupational risk factor exposure	26 (7.8)	7 (16.7)	19 (6.5)	0.058		
Previous cancer history	16 (4.8)	4 (9.5)	12 (4.1)	0.257		
Diabetes mellitus	52 (15.5)	11 (26.2)	41 (14.0)	0.080		
Systemic inflammatory disease	70 (20.9)	9 (21.4)	61 (20.8)	0.407		
Metabolic syndrome	90 (26.9)	16 (38.1)	74 (25.3)	0.436		
Immunosuppressive regimen^a^						
Cyclosporine	254 (75.8)	39 (92.9)	215 (73.4)	**0.037**		
Tacrolimus	75 (23.7)	3 (7.1)	72 (24.6)	**0.006**		
Mycophenolic acid	256 (76.4)	31 (73.8)	225 (76.8)	0.150		
Everolimus	134 (40.0)	21 (50.0)	113 (38.6)	0.312		
Exposure to immunosuppressive agent, days						
Cyclosporine	2794 [728.5–4645.0]	2320 [1620–3558]	2871 [370–4894]	0.570		
Tacrolimus	2075 [817–4477]	1206 [1103–1206]	2093 [710–4512]	0.508		
Mycophenolic acid	1728 [186–3716]	1781 [663–2616]	1712 [168–3897]	0.684		
Everolimus	3087 [766–4615]	1632 [771–3678]	3433 [740–4888]	0.116		
Pre‐transplant PRA positivity	62 (18.5)	5 (11.9)	57 (19.4)	0.256		
Acute cellular rejection	77 (23)	11 (26.2)	66 (22.5)	0.676		
Antibody‐mediated rejection	43 (12.8)	5 (11.9)	38 (13.0)	1.000		
Cardiac allograft vasculopathy	50 (14.9)	3 (7.1)	47 (16.0)	0.161		
Donor‐specific antibodies	48 (14.3)	7 (16.7)	41 (14.0)	0.820		
Time interval transplantation‐last follow up, days	2817 [339–4788]	3561 [2457–5539]	2747 [166–4655]	**0.002**	1.02 [1.00–1.04]^b^	**0.007**
Mismatch CMV serology (D^+^R^–^)	56 (16.7)	10 (23.8)	46 (15.6)	0.167		
CMV reactivation	212 (63.3)	32 (76.2)	180 (61.4)	0.869		
EBV reactivation	118 (35.2)	15 (35.7)	103 (35.2)	0.982		
Time interval cancer occurrence‐last follow‐up, days	657 [288–1876]					
Time interval HTx‐cancer occurrence, days	2303 [1496–3509]					

*Note:* Data are *N* (%) or median [IQR]. In bold *p* values < 0.05.

Abbreviations: CMV, cytomegalovirus; EBV, Epstein‐Barr virus; HTx, heart transplantation; PRA, Panel Reactive Antibody.

^a^ Twenty‐two patients died in the early post‐transplant period and no data about immunosuppressive regimen were available. One patient did not have a Calcineurin inhibitor regimen. In addition, this value represents all patients that have received, even for a limited time post‐transplant, each immunosuppressive agent.

^b^ Due to the high values of these numbers, in the multivariable analysis every count of the variable was divided by 100.

Among patients who developed cancer, the median time interval between HTx and cancer diagnosis was 2303 days [IQR: 1496–3509] corresponding to about 6.3 years. Median survival from cancer diagnosis to the end of follow‐up was 657 days [IQR: 288–1876].

**FIGURE 1:**
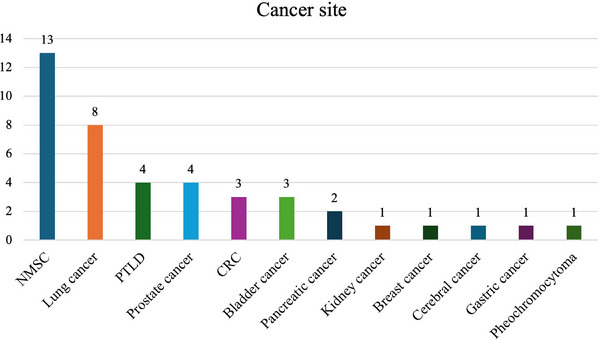
Cancer sites distribution. CRC, colorectal cancer; NMSC, non‐melanoma skin cancer; PTLD, post‐transplant lymphoproliferative disorders.

The most common malignancies were non‐melanoma skin cancer (NMSC) (13 cases, 31%) and lung cancer (8 cases, 19%). Among non‐melanoma skin cancers, 9 (69.2%) and 4 (30.8%) were basal cell and squamous cell carcinomas, respectively (details in Figure 1).

Based on the occurrence of cancer, patients were divided in two groups. At univariable analysis, those who developed cancer were older at HTx (57 vs. 52 years, *p* = 0.006), and were more frequently men (90.5% vs. 75.8%, *p* = 0.031). Cancer onset was also associated with cigarette smoking (78.6% vs. 47.4%, *p* = 0.011) and alcohol consumption (42.9% vs. 22.5%, *p* = 0.043). Ischemic heart disease was the indication for HTx more often among recipients who developed cancer (61.9% vs. 33.1%, *p* < 0.001); furthermore, patients with a diagnosis of cancer had a longer length of follow‐up (3561 vs. 2747 days, *p* = 0.002). Immunosuppressive regimens differed between the two groups; in particular, patients who developed cancer had been more frequently prescribed cyclosporine (92.9% vs. 73.4%, *p* = 0.037) and less frequently tacrolimus (7.1% vs. 24.6%, *p* = 0.006), even if no differences emerged analyzing the days of exposure to each immunosuppressant. However, median age was significantly higher in those taking cyclosporine (54 [47.0–59.5] vs. 42 [29.5–57] years; *p* < 0.001).

No differences were observed between groups with and without cancer for any type of graft rejection, virus reactivation, and other comorbidities (Table 1). Based on the number of patients in the index group (cancer group = 42 patients), we entered five variables in the multivariable analysis. Prior ischemic heart disease (OR 2.70, 95% CI 1.19–6.11, *p* = 0.017) and length of follow‐up (OR 1.02, 95% CI 1.00–1.04, *p* = 0.007) were independent predictors of cancer occurrence. All analyses are shown in Table 1. Kaplan Meier curve for cancer occurrence according to the year of HTx did not show differences among groups (log rank test *p* = 0.09), Figure S3.

**FIGURE 2:**
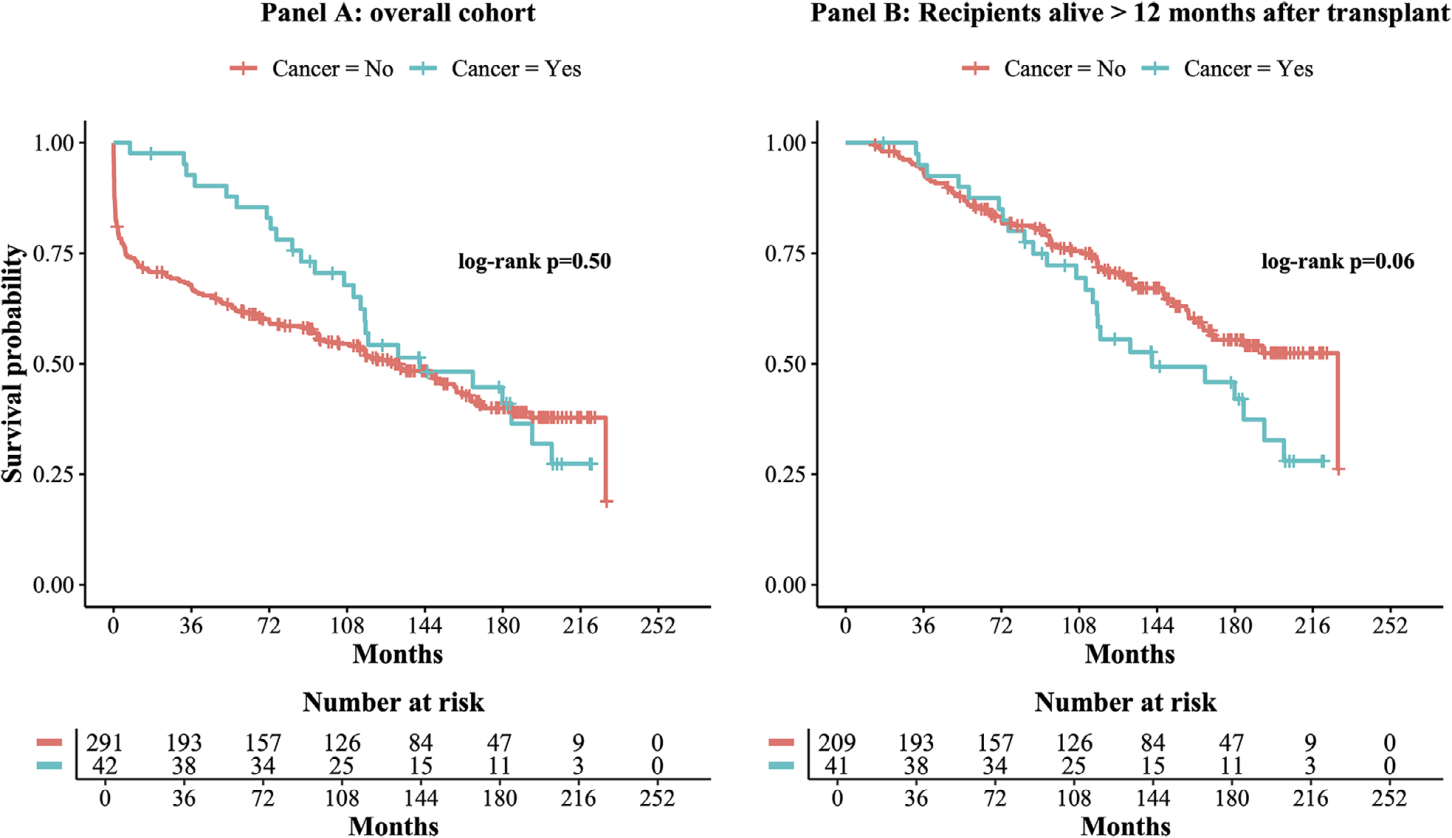
Kaplan Meier survival curves of the entire cohort (Panel A) and of patients with a follow‐up >12 months (Panel B). In light blue patients with a cancer diagnosis during follow‐up. In red all other patients.

Cox regression for mortality using cancer occurrence as a time varying covariate and adjusted for sex, age, ischemic cardiomyopathy as cause of transplant, smoking history and diabetes showed a higher risk of mortality among HTx recipients who developed cancer during follow‐up (HR 3.400, 95% C.I. [2.026–5.709] *p* < 0.001), Table 2. Kaplan Meier analysis showed a trend for a different survival between the two groups after exclusion of patients with a follow‐up shorter than 1 year (log rank *p* = 0.06, Figure 2).

**TABLE 2:** Time‐varying Cox regression for all‐cause mortality.

Risk factor for mortality	Cox regression with time varying hazard model, HR [95% CI]	*p* value
Cancer occurrence	3.400 [2.026–5.709]	**<0.001**
Sex, *male*	1.112 [0.639–1.934]	0.708
Age at HTx	1.020 [1.000–1.040]	0.053
Prior ischemic heart disease	1.076 [0.686–1.686]	0.751
Smoking history	0.804 [0.465–1.389]	0.434
CMV reactivation	0.781 [0.490–1.246]	0.300
Acute cellular rejection	1.036 [0.654–1.641]	0.879
Diabetes mellitus	1.376 [0.854–2.219]	0.190
Antibody‐mediated rejection	1.579 [0.971–2.567]	0.066

In bold *p* value <0.05.

Abbreviation: HTx, heart transplantation.

Among patients with a previous cancer history, only two had cancer after HTx, of whom one developed a B‐cell lymphoma recurrence and one a de novo bladder cancer (data not shown).

Clinical characteristics of patients with lung cancer and NMSC compared with patients without malignancies are shown in Tables S1 and S2. Among lung cancer patients, age, family history, alcohol consumption, and cigarette smoking were associated with cancer occurrence. Of interest, a statistical trend was observed for ischemic heart disease as indication for HTx (Table S1). Lung cancer diagnosis at Stages I, III, and IV occurred in three, one, and four patients, respectively. Patients with NMSC also presented more frequently ischemic cardiomyopathy as the indication for transplant. The Kaplan Meier survival analysis according to lung cancer occurrence showed a dramatic reduction of survival in affected recipients (log rank *p* = 0.001, Figure S1). On the other hand, no differences were found according to NMSC occurrence (log rank *p* = 0.168, Figure S2).

## 4 Discussion

In this single center experience, we analyzed clinical characteristics and risk factors for cancer in HTx recipients followed up in our hospital. The absolute prevalence of cancer in our cohort was lower compared with previous studies, likely due to a shorter follow up time in our cohort [7, 8, 16].

The median time between HTx and cancer diagnosis was >6 years, similar to earlier studies [7]. The most common cancer was NMSC. At variance with the general population, the occurrence of squamous cell carcinoma is usually higher than basal cell carcinoma in solid organ transplant recipients [17, 18, 19, 20, 21]. However, in our cohort, basal cell carcinoma accounted for 69.2% of NMSC, consistent with another Italian cohort, where a 2.1:1 basal cell: squamous cell carcinoma ratio was reported [22]. Lung cancer was the most common solid tumor among HTx recipients and in our study accounted for 19% of cases, the second most frequent cancer [8]. This might be explained by the shared risk profile between some HTx indications and lung cancer, including smoking history [23].

Indeed, together with higher age at transplantation, male sex, alcohol consumption, and use of cyclosporine instead of tacrolimus, smoking history was associated with cancer. A higher age at transplant in patients with cancer occurrence could be explained by the usually higher age of HTx recipients with ischemic heart disease [24]. In addition, a role may be played by mechanisms that link senescence and cancer [25, 26, 27, 28].

As in the general population, patients with cancer were more frequently male [29]. Possible mechanisms are hormone expressions, genetic predisposition, metabolism, immune system, and exposure to carcinogens such as alcohol and cigarette smoking, the latter more represented among males for cultural and historical reasons [30, 31, 32, 33, 34, 35].

Alcohol consumption was also associated with cancer at univariate analysis. Mechanisms involved may be different, including production of reactive oxygen species (ROS) and pro‐inflammatory cytokines, interaction with folates and retinoid metabolism, and reduced immune function [36]. Alcohol relation with liver, colorectal, gastric, pancreatic, prostate, and breast cancers is well known, although the association with NMSC and lung cancer (that in our cohort accounted for more than half of patients) is still controversial [37, 38]. Whether alcohol may act enhancing chronic immunosuppression and reducing immune system control on cancer transformation in HTx recipients remains to be assessed.

Calcineurin inhibitors are the cornerstone of immunosuppressive therapy in HTx recipients [3]. Among those who developed cancer, a more frequent use of cyclosporine and a lesser use of tacrolimus were observed, in line with previous findings [29]. Xu et al. found increased phosphorylation of transforming growth factor β‐activating kinase 1 (TAK1) and its binding with TAB1 and TAB2, secondary to cyclosporin use. These pathways increase activation of p38, NFkB, and MAP‐kinase, leading to tumorigenesis in squamous cell carcinoma [39]. We believe that caution should be used in interpreting these data since the association between cyclosporine and cancer disappeared when total exposure to each immunosuppressive agent was taken into account. A lower use of tacrolimus in patients with cancer may also be explained by the lower median age of patients who received this molecule. On the other hand, Molina et al. reported a lower incidence of skin cancer in patients that used tacrolimus during the first 3 months after transplant, even if the correlation was lost at multivariable analysis and no underlying mechanisms were identified [17].

All included patients received steroids and only three did not receive anti‐thymocyte globulin (ATG), making impossible to assess the effect of induction therapy on cancer development.

At multivariable analysis, history of prior ischemic cardiomyopathy emerged in our cohort as an independent predictor of cancer during follow‐up, in line with previous literature and independent of cigarette smoking in this subgroup (*p* < 0.001, data not shown) [29]. Previous studies showed the same result in non‐HTx populations and in larger cohorts [40]. In addition, Hasin et al. showed that the presence of heart failure after myocardial infarction correlated with cancer occurrence [41]. Chronic inflammation secondary to obesity and hypertension, which usually prevail in ischemic cardiomyopathy populations, may partially explain the higher risk of cancer development in these patients [42, 43, 44, 45, 46].

In our study, length of follow‐up independently associated with cancer occurrence, leading to a possible misinterpretation that cancer patients have a better survival after HTx. Certainly, a role is played by a more prolonged exposure to immunosuppression [47].

To assess the impact of cancer on survival after HTx, limiting the immortal‐time bias, we performed a Cox regression using cancer occurrence during follow‐up as a time varying covariate, and observed a 3.4 times higher rate of mortality at any time in patients with cancer occurrence. Kaplan Meier analysis of the overall cohort failed to show a statistically significant difference in survival between patients with and without cancer during follow‐up, even if graphical differences were noted. To reduce the impact of immortal‐time bias, we subsequently excluded from the analysis patients with a follow‐up shorter than 1‐year, considering at least that time period is needed to develop any cancer. Interestingly, in this subgroup, differences were more evident, but only a statistical trend was observed at log‐rank test.

Additionally, survival differed according to cancer site. As a matter of fact, NMSC had better prognosis compared to lung cancer. On the other hand, recipients who developed lung cancer had very few differences compared to the remaining cohort (Table S1). In particular, a higher age at transplant, smoking history, alcohol use and family history of cancer were more frequent in patients with lung cancer. Of note, only a statistical trend was observed for ischemic cardiomyopathy as a cause of transplant (Table S1). Finally, this subgroup showed a dismal prognosis (100% mortality during follow‐up) possibly due to the advanced stage of the disease. Indeed, 5 of 8 patients (62.5%) had a Stage III or IV disease at diagnosis. Lung cancer occurrence among HTx patients was associated with worse outcomes even when the disease was diagnosed at an early stage [48].

No analyzed risk factors specifically correlated with NMSC, the most frequent cancer observed in our study, with the exception of a higher prevalence of ischemic cardiomyopathy as the indication for transplant (Table S2). Even if this correlation was previously reported, underlying mechanisms remain to be elucidated [27].

Relationship of EBV infection with post‐transplant lymphoproliferative disorders is well defined and guidelines assessing management of EBV infection in patients with PTLD have been published [49]. In our cohort, EBV infection/reactivation did not correlate with cancer occurrence. The low number of PTLD cases (four patients) and the frequent finding of EBV reactivation/infection regardless of viral load may explain this result.

Limitations of our study were the low number of patients in the cancer group and missing data due to the retrospective nature of study design.

Additional studies are surely needed to better understand the mechanisms and risk factors for cancer in HTx recipients to improve prevention and early diagnosis among patients at higher risk.

In conclusion, HTx recipients are at high risk for cancer development. Although there are, to date, no differences in oncology screening programs between HTx recipients and the general population, our data suggest efforts should be focused on patients with a prior history of ischemic heart disease and those with a longer survival time after transplantation.

## Author Contributions

Fabian Patauner, Rosa Zampino, and Emanuele Durante‐Mangoni participated in research design, drafting, and critical revision of the paper. Filomena Boccia, Silvia Masini, Giuliana Autiero, Irene Mattucci, Daniela Pinto, and Roberto Andini participated in data collection. Cristiano Amarelli participated in research design. Raffaella Gallo and Lorenzo Bertolino participated in data analysis and statistics.

## Conflicts of Interest

E.D.M. received institutional funding, personal speaker fees, or advisory board membership honoraria from Pfizer, Angelini, Advanz pharma, Infectopharm, Shionogi, Menarini, Abbvie, Trx, outside of this work. R.Z. received grant support and personal fees from Gilead Sciences, Nordic Pharma, Ikos, outside of this work. The other authors declare no conflicts of interest.

## Supporting information




**Supplementary Table 1:** Analysis of factors associated with lung cancer development in heart transplant recipients.
**Supplementary Table 2:** Analysis of factors associated with non‐melanoma skin cancer occurrence in heart transplant recipients.
**Supplementary Table 3:** Time interval between HTx and cancer occurrence according to Cancer site.
**Supplementary Figure 1:** Kaplan Meier survival curves of patients with a follow‐up >12 months. In light blue patients with a lung cancer diagnosis during follow‐up. In red patients who did not develop any cancer during follow up.
**Supplementary Figure 2:** Kaplan Meier survival curves of patients with a follow‐up >12 months. In light blue patients with a NMSC diagnosis during follow‐up. In red patients who did not develop any cancer during follow up. NMSC, non‐melanoma skin cancer.
**Supplementary Figure 3:** Kaplan Meier curves for cancer occurrence according to the year of transplant. In red, green, light green, blue and purple, patients who underwent heart transplantation in 2005–2007, 2008–2010, 2011–2013, 2014–2016, 2017–2019, respectively. HTx, heart transplantation.

## Data Availability

The data that support the findings of this study are available from the corresponding author upon reasonable request.
