# Supplementary material for: Conditions Associated With the Onset of Cancer After Heart Transplant: Longitudinal Study in 335 Recipients
Source: Clin Transplant. 2025 Jul 25;39(8):e70243. doi: 10.1111/ctr.70243 (PMC12290768; doi:10.1111/ctr.70243)
Supplement: Supplementary file 1 — Supplementary Table 1: Analysis of factors associated with lung cancer development in heart transplant recipients. Supplementary Table 2: Analysis of factors associated with non‐melanoma skin cancer occurrence in heart transplant recipients. Supplementary Table 3: Time interval between HTx and cancer occurrence according to Cancer site. Supplementary Figure 1: Kaplan Meier survival curves of patients with a follow‐up >12 months. In light blue patients with a lung cancer diagnosis during follow‐up. In red patients who did not develop any cancer during follow up. Supplementary Figure 2: Kaplan Meier survival curves of patients with a follow‐up >12 months. In light blue patients with a NMSC diagnosis during follow‐up. In red patients who did not develop any cancer during follow up. NMSC, non‐melanoma skin cancer. Supplementary Figure 3: Kaplan Meier curves for cancer occurrence according to the year of transplant. In red, green, light green, blue and purple, patients who underwent heart transplantation in 2005–2007, 2008–2010, 2011–2013, 2014–2016, 2017–2019, respectively. HTx, heart transplantation. [file CTR-39-e70243-s001.docx]

| **Supplementary Table 1. Analysis of factors associated with lung cancer development in heart transplant recipients** | | | |
| --- | --- | --- | --- |
|  | **Lung cancer** **N=8** | **No Lung Cancer** **N=327** | **p value** |
| Age at HTx | 58.5 [54-65] | 52 [42-59] | **0.034** |
| Sex, *male* | 8 (100) | 252 (77.1) | 0.207 |
| Family history of cancer | 5 (62.5) | 51 (15.6) | **0.014** |
| Smoking history | 8 (100) | 164 (50.2) | **0.007** |
| Alcohol exposure | 7 (87.5) | 77 (59.3) | **0.010** |
| Occupational exposure | 2 (25) | 24 (7.3) | 0.207 |
| Previous cancer history | 0 (0) | 16 (4.9) | 1.000 |
| Diabetes mellitus | 2 (25.0) | 50 (15.3) | 0.630 |
| Metabolic syndrome | 1 (12.5) | 89 (27.2) | 0.651 |
| Cellular mediated rejection | 2 (25) | 75 (22.9) | 1.000 |
| Antibody-mediated rejection | 0 (0) | 43 (12.8) | 1.000 |
| Cardiac allograft vasculopathy | 1 (12.5) | 49 (15) | 1.000 |
| Immunosuppressive regimen |  |  |  |
| *Cyclosporine* | 7 (87.5) | 247 (75.5) | 1.000 |
| *Tacrolimus* | 1 (12.5) | 74 (22.6) | 0.685 |
| *Mycophenolic acid* | 8 (100) | 248 (75.8) | 0.359 |
| *Everolimus* | 3 (37.5) | 131 (40.0) | 1.000 |
| Exposure to immunosuppressive agent, days |  |  |  |
| *Cyclosporine* | 2882 [1936-3031] | 2780 [525-4663] | 0.944 |
| *Tacrolimus* | 1103 [1103-1103] | 2093 [739-4484] | 0.456 |
| *Mycophenolic acid* | 1743 [1203-2993] | 1728 [166-3804] | 0.644 |
| *Everolimus* | 1001 [898-] | 3218 [688-4629] | 0.206 |
| Ischemic cardiomyopathy as cause of transplant | 6 (75) | 117 (35.8) | 0.055 |
| Data are N (%) or median [IQR]  *HTx, Heart transplantation;* | | | |

| **Supplementary Table 2. Analysis of factors associated with non-melanoma skin cancer occurrence in heart transplant recipients** | | | |
| --- | --- | --- | --- |
|  | **NMSC N=13** | **No NMSC N=322** | **p value** |
| Age at HTx | 57 [52-60] | 52 [41-59] | 0.078 |
| Sex, male (%) | 12 (92.3) | 248 (77.0) | 0.312 |
| Family history of cancer | 4 (30) | 52 (16.1) | 0.244 |
| Smoking history | 9 (69.2) | 163 (50.6) | 0.291 |
| Alcohol exposure | 4 (34) | 80 (24.8) | 0.453 |
| Occupational exposure | 1 (7.6) | 25 (7.7) | 0.526 |
| Previous cancer history | 0 (0) | 16 (4.9) | 1.000 |
| Diabetes mellitus | 2 (15.4) | 50 (15.5) | 1.000 |
| Metabolic syndrome | 7 (53.8) | 83 (25.7) | 0.257 |
| Cellular mediated rejection | 4 (34) | 73 (22.6) | 0.501 |
| Antibody-mediated rejection | 2 (15.3) | 41 (12.7) | 0.653 |
| Cardiac allograft vasculopathy | 1 (7.6) | 49 (15.2) | 0.308 |
| Immunosuppressive regimen |  |  |  |
| *Cyclosporine* | 11 (84.6) | 243 (75.4) | 1.000 |
| *Tacrolimus* | 2 (15.3) | 73 (22.6) | 0.740 |
| *Mycophenolic acid* | 9 (69.2) | 247 (76.7) | 0.265 |
| *Everolimus* | 8 (61.5.0) | 126 (39.1) | 0.163 |
| Exposure to immunosuppressive agent, days |  |  |  |
| *Cyclosporine* | 3460 [2060-4394] | 2780 [499-4663] | 0.406 |
| *Tacrolimus* | 1784 [1206-] | 2075 [724-4498] | 0.784 |
| *Mycophenolic acid* | 1206 [118-3385] | 1740 [192-3740] | 0.788 |
| *Everolimus* | 2761 [1739-3896] | 3087 [662-4689] | 0.939 |
| Ischemic cardiomyopathy as indication for transplant | 9 (69.2) | 114 (35.4) | **0.018** |
| Data are N (%) or median [IQR]  *HTx, Heart transplantation;* |  |  |  |

| **Supplementary Table 3. Time interval between HTx and cancer occurrence according to Cancer site** | |
| --- | --- |
| Cancer site | Time Interval HTx-Cancer occurrence, days |
| NMSC | 2362 [1846 -4268] |
| Lung cancer | 2423 [1626-3031] |
| PTLD | 1810[703-2204] |
| Prostate cancer | 3298 [1123-3910] |
| CRC | 2510 |
| Bladder cancer | 1404 |
| Pancreatic cancer | 2534 |
| Kidney cancer | 2583 |
| Breast cancer | 2593 |
| Cerebral cancer | 1770 |
| Gastric cancer | 1573 |
| Pheochromocytoma | 26 |
| Data are median [IQR], for cancer sites counting ≤ 3 cases only median is reported.  *CRC, Colorectal cancer; HTx, Heart transplantation; NMSC, Non Melanoma Skin Cancer; PTLD, post-transplant lymphoproliferative disorders* | |
|  | |


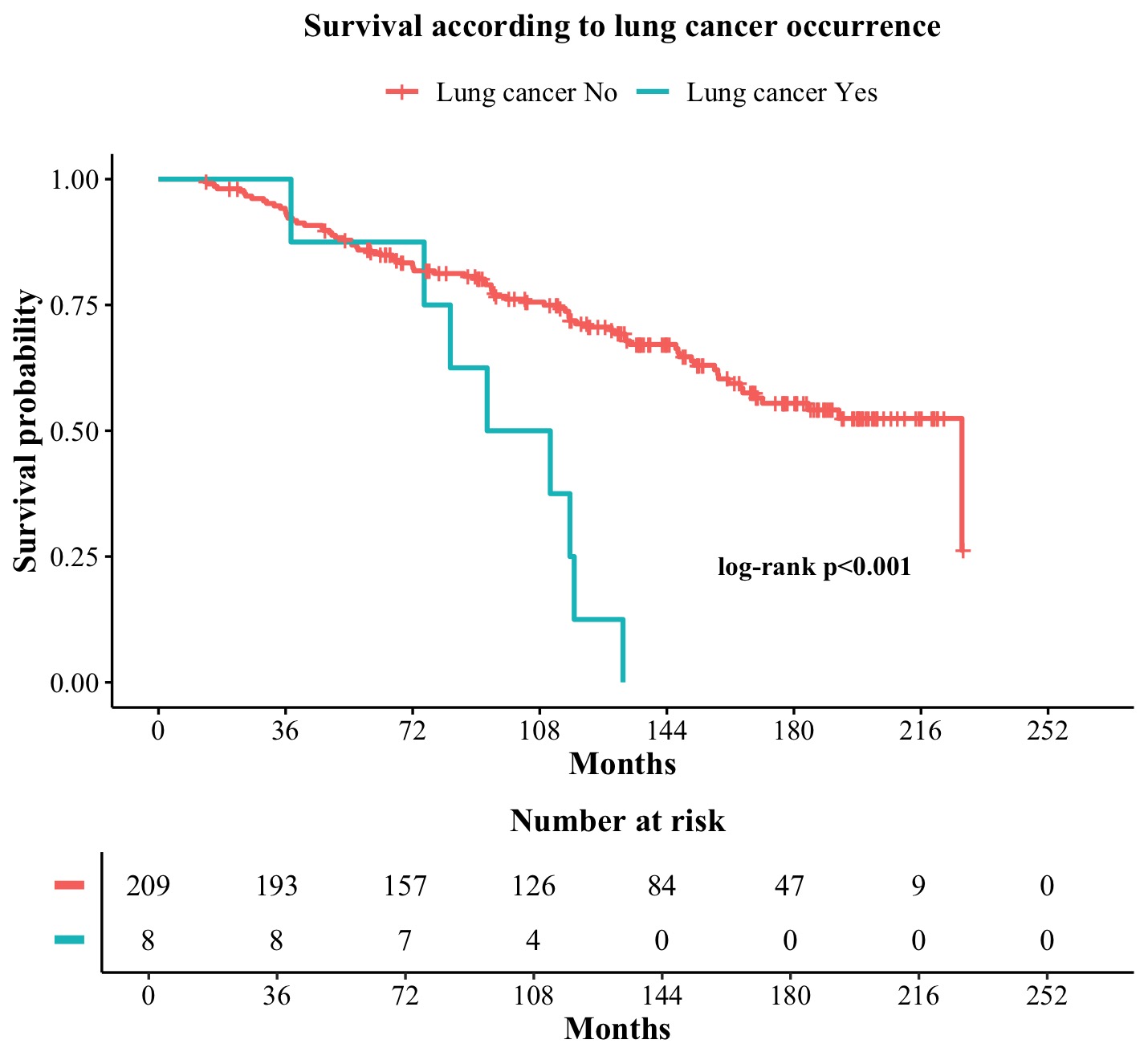


**Supplementary Figure 1.** Kaplan Meier survival curves of patients with a follow-up >12 months. In light blue patients with a lung cancer diagnosis during follow-up. In red patients who did not develop any cancer during follow up.


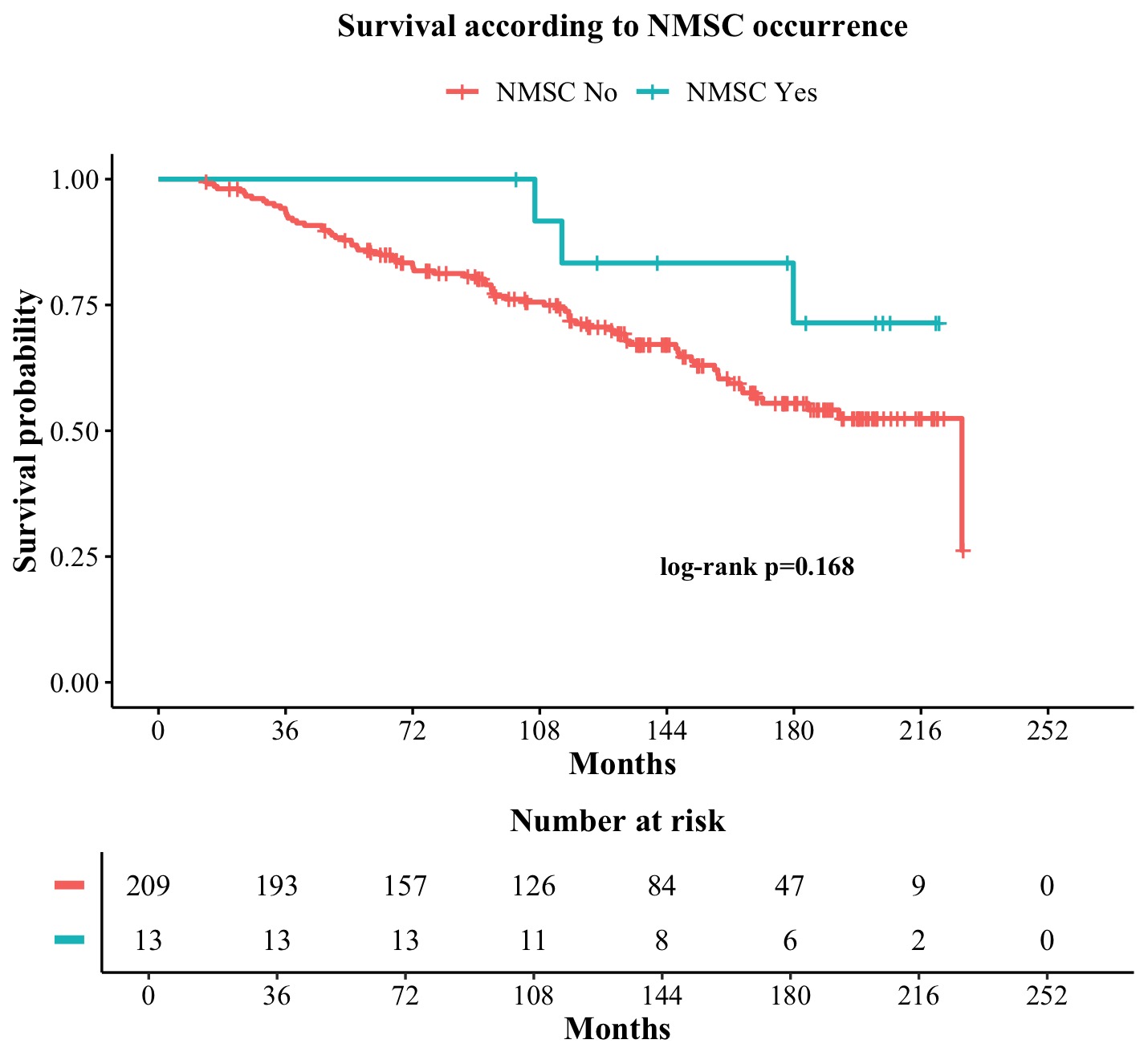


**Supplementary Figure 2.** Kaplan Meier survival curves of patients with a follow-up >12 months. In light blue patients with a NMSC diagnosis during follow-up. In red patients who did not develop any cancer during follow up. NMSC, non-melanoma skin cancer.


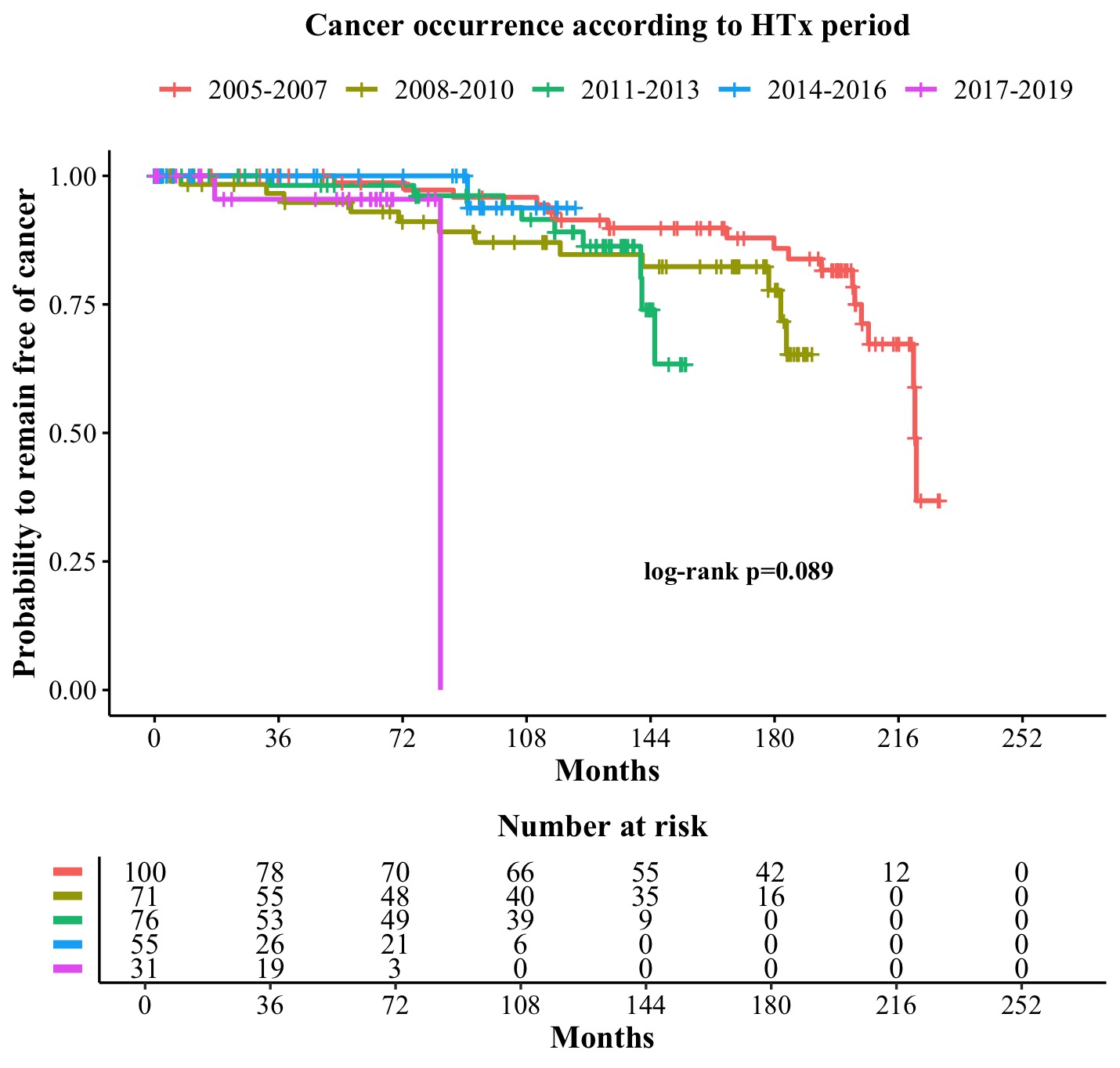


**Supplementary Figure 3.** Kaplan Meier curves for cancer occurrence according to the year of transplant. In red, green, light green, blue and purple, patients who underwent heart transplantation in 2005-2007, 2008-2010, 2011-2013, 2014-2016, 2017-2019, respectively. HTx, heart transplantation.
